# Alternative Splicing of Differentiated Myeloid Cell Transcripts after Infection by *Anaplasma phagocytophilum* Impacts a Selective Group of Cellular Programs

**DOI:** 10.3389/fcimb.2018.00014

**Published:** 2018-02-02

**Authors:** J. Stephen Dumler, Sara H. Sinclair, Amol C. Shetty

**Affiliations:** ^1^Department of Pathology, F. Edward Hébert School of Medicine, Uniformed Services University of the Health Sciences, Bethesda, MD, United States; ^2^Independent Researcher, Tsaile, AZ, United States; ^3^Institute for Genome Sciences, University of Maryland, Baltimore, Baltimore, MD, United States

**Keywords:** *Anaplasma phagocytophilum*, intracellular infection, RNA isoforms, RNAseq, spliceosome, gene ontology

## Abstract

Eukaryotic proteome diversity exceeds that encoded within individual genes, and results in part from alternative splicing events of pre-messenger RNA. The diversity of these splicing events can shape the outcome in development and differentiation of normal tissues, and is important in pathogenic circumstances such as cancer and some heritable conditions. A role for alternative splicing of eukaryotic genes in response to viral and intracellular bacterial infections has only recently been recognized, and plays an important role in providing fitness for microbial survival, while potentially enhancing pathogenicity. *Anaplasma phagocytophilum* survives within mammalian neutrophils by reshaping transcriptional programs that govern cellular functions. We applied next generation RNAseq to ATRA-differentiated HL-60 cells established to possess transcriptional and functional responses similar to *A. phagocytophilum*-infected human neutrophils. This demonstrated an increase in transcripts with infection and high proportion of alternatively spliced transcript events (ASEs) for which predicted gene ontology processes were in part distinct from those identified by evaluation of single transcripts or gene-level analyses alone. The alternative isoforms are not on average shorter, and no alternative splicing in genes encoding spliceosome components is noted. Although not evident at gene-level analyses, individual spliceosome transcripts that impact nearly all spliceosome components were significantly upregulated. How the distinct GO processes predicted by ASEs are regulated by infection and whether they are relevant to fitness or pathogenicity of *A. phagocytophilum* should be addressed in more detailed studies.

## Introduction

The small genomes of many obligate intracellular bacteria provide a conundrum as to the limited genomic resources required to promote fitness in the environment of a host cell with a genome reservoir thousands times greater. Recent years have demonstrated the interplay between the genomic products of intracellular bacteria and those of their host cells, including an increasing body of literature implicating direct action on the host genome by microbial effectors (Bierne and Cossart, 2012; Sinclair et al., 2014; Prokop et al., 2017). Such is the case with the tick-transmitted obligate intracellular rickettsia, *Anaplasma phagocytophilum* (Garcia-Garcia et al., 2009a,b; Rennoll-Bankert et al., 2015; Sinclair et al., 2015; Dumler et al., 2016). While its reserves of effectors are multiple and many have moonlighting functions (Lin et al., 2007; Truchan et al., 2013), the extent of neutrophil reprogramming that impacts bacterial fitness after infection is difficult to explain (Carlyon et al., 2002; Choi and Dumler, 2003; Choi et al., 2003, 2004a, 2005; Park et al., 2003; Garyu et al., 2005; Carlyon and Fikrig, 2006). Chromatin reconfiguration and transcriptional reprogramming under the control of microbial effectors, including AnkA, demonstrate that the extended genome of *A. phagocytophilum* includes those targets in the genome of the host cell as well.

*A. phagocytophilum* reprogramming of specific functions, such as respiratory burst driven by AnkA recruitment of HDAC-1 to the promoter of *CYBB* is an example of *cis*-gene silencing by restructuring of chromatin histone H3 at the gene promoter (Garcia-Garcia et al., 2009a,b; Rennoll-Bankert et al., 2015), yet global reprogramming becomes a greater challenge to explain. Although AnkA binds genomic sites broadly across every human chromosome in human model systems, a direct link between AnkA binding and transcriptional program changes is still not well-investigated (Park et al., 2004; Dumler et al., 2016). Stronger candidates for transcriptional and functional reprogramming include the sequestration of AnkA-bound DNA into the nuclear lamina that reshapes nuclear architecture and potentially cellular programs, and the magnitude of host cell DNA methylation after infection across all genomic features which provide opportunities for further exploration (Sinclair et al., 2015; Dumler et al., 2016). While transcriptional regulation is governed by many factors, the RNA landscape of cells plays a major role in events such as induced pluripotent stem cell reprogramming (Gamazon and Stranger, 2014), cellular differentiation (Fiszbein and Kornblihtt, 2017; Keightley and Lieschke, 2017), and oncogenesis (Narayanan et al., 2017). In fact, the RNA landscapes before and after infection by viruses and bacteria such as *Mycobacterium tuberculosis* and *Mycoplasma pneumoniae* demonstrate a role for alternative transcript splicing events as key fitness determinants that regulate intracellular survival and transmission (Akusjarvi, 2008; Boudreault et al., 2016; Hu et al., 2016; Graham and Faizo, 2017; Kalam et al., 2017; Wang et al., 2017). While methylated DNA in exons is well-known to play a role in alternative splicing events (ASEs), a role for this in infections has not been examined (Shukla et al., 2011; Maunakea et al., 2013; Lev Maor et al., 2015).

In this work, we interrogate a model of all-trans retinoic acid (ATRA)-differentiated HL-60 cells infected by *A. phagocytophilum* that we previously demonstrated to have transcriptional profiles most closely similar to *ex vivo* human neutrophils (Rennoll-Bankert et al., 2014), and demonstrate that ASEs occur in 18% of over 600 differentially expressed transcripts. Gene ontology processes enriched within this subset of genes that undergo alternative splicing map to unique pathways not identified by gene-level analyses. The lack of marked changes in alternative splicing among spliceosome genes as observed with *M. tuberculosis* infection of macrophages, and the lack of a significant change in overall transcript size among ASEs as observed with viral infection demonstrate that *A. phagocytophilum* infection is associated with a distinct profile of ASEs. These findings provide additional support for the role that alternative splicing plays in infection and microbial fitness within intracellular niches, and provides another example of complexity in how microbes regulate host gene expression via alternative splicing.

## Materials and methods

### *A. phagocytophilum* infection in ATRA-differentiated HL-60 cell model

We used the model as we previously described (Rennoll-Bankert et al., 2014). Briefly, the human promyelocytic HL-60 (ATCC CCL-240) cell line was purchased from American Type Culture Collection (Manassas, VA). HL-60 cells were differentiated 5 days with 1 μM ATRA prior to infection. Cells were grown in a humidified incubator at 37°C with 5% CO_2_. Cell density was kept <10^6^ cells mL^−1^ by diluting with fresh medium. Infection was established by inoculating low passage (<10 passages *in vitro*) *A. phagocytophilum* (Webster strain^T^)-infected HL-60 cells into freshly prepared HL-60 cells to contain ~20% infected cells. After infection was established, the proportion of infected cells was adjusted to 10–20% with uninfected HL-60 cells and ATRA was added to the medium. After 5 days, triplicate cultures that contained >90% infected cells and triplicate uninfected cultures were harvested. RNA was prepared using the Zymo Quick-RNA miniprep (Irvine, CA) kit. Control ATRA-differentiated HL-60 cells were maintained in parallel but uninfected.

### TruSeq RNA-Seq libraries, and illumina HiSeq2000 sequencing

Illumina RNA-Seq libraries were prepared with the TruSeq RNA Sample Prep kit (Illumina, San Diego, CA) per manufacturer's protocol. Adapters containing six nucleotide indexes were ligated to the double-stranded cDNA. The DNA was purified between enzymatic reactions and library size selection was performed with AMPure XT beads (Beckman Coulter Genomics, Danvers, MA). Libraries were multiplexed in two groups of three per flowcell lane using a 100 bp paired-end run.

### RNAseq alignment and visualization

The RNAseq alignment and visualization pipeline used the FastX-toolkit (http://hannonlab.cshl.edu/fastx_toolkit/) for quality control and read trimming. Subsequently, short RNAseq reads were aligned using TopHat, a splice-aware aligner which is specifically built upon the Bowtie short read aligner for eukaryotic genomes (Trapnell et al., 2009; Langmead, 2010; Langmead and Salzberg, 2012) against the GRCh37 human genome.

### RNAseq differential expression analysis

The pipeline output was used to perform differential gene expression by fold-change calculations on normalized RPKM (Mortazavi et al., 2008) (reads per kilobase per million mapped reads) values to measure gene level expression or FPKM (fragments per kilobase per million) values to measure isoform level expression. We used Cuffdiff (Trapnell et al., 2010) to identify differentially expressed genes or isoforms under infected and uninfected conditions. To compare results with previous expression profiling studies of *A. phagocytophilum* infection in human myeloid cells and primary neutrophils, differentially expressed genes from five other studies (Borjesson et al., 2005; de la Fuente et al., 2005; Pedra et al., 2005; Lee and Goodman, 2006; Lee et al., 2008), all conducted using microarray technologies, were compared for shared differentially regulated genes and for GO processes (Gene Ontology enRIchment anaLysis and visuaLizAtion tool [Gorilla] http://cbl-gorilla.cs.technion.ac.il/) (Eden et al., 2009) predicted based on those differentially regulated genes. Comparisons were visualized by creating Venn diagrams (http://bioinforx.com/free/bxarrays/venndiagram.php) using all differentially-expressed genes identified at *p*-values below 0.05 and absolute log_2_ fold change >1, and by comparing GO processes numbers identified when analyzing each of the sets of differentially expressed genes.

### Analysis of alternative splicing

ASEs were identified by comparison of individual transcripts to gene records, and filtered to keep only isoform data with at least two replicates for both *A. phagocytophilum*-infected ATRA-differentiated HL-60 cells and uninfected cells. Events with a *p*-value < 0.05 were retained. To ensure higher stringency, the ASEs were further filtered with a cutoff *Q*-value < 0.05 and fold-change in isoform transcription between infected and uninfected conditions greater than 2 to obtain a total initial set. Further filtering to assure sufficient transcript number included only isoforms for which FPKM was >1; these were evaluated for percent spliced-in (PSI) metrics, as described previously (Wang et al., 2008; Boudreault et al., 2016). In brief, PSI estimates diversity of transcript isoforms (to characterize inclusion of exon, differential splice-site choice, intron retention, etc.) for a single gene as a function of the longest isoform/longest + shorter isoforms (ψ = L/(L+S)). From these events, only those with a difference higher than 7.5% in PSI among infected cells were considered biologically relevant.

### Analysis of changes in spliceosome-associated isoform expression

To analyze the participation of regulated expression of spliceosome components on alternative splicing events, a list of genes involved in splicing events was downloaded from the spliceosome database (http://spliceosomedb.ucsc.edu). This list included 135 genes involved in splicing that were used to query the differentially transcribed genes and identified by RNAseq. The spliceosome genes were then further queried to identify whether other isoform variants were differentially expressed in either infected of uninfected cells, and to determine whether the isoforms identified were considered the principal variant, or one of several alternative splicing events that could impact expression and therefore splicing events.

### Gene ontology analysis of gene-level and transcript isoform-level expression

In order to discern specific pathways enriched with infection, Gene Ontology analysis was conducted using the gene-level differentially expressed genes, using genes identified by all isoforms, and genes with alternatively spliced isoforms that were significantly expressed. The GO analyses were conducted using Gorilla, as above, and for specific ASE isoforms, Panther GO was used (http://www.pantherdb.org/). For gene-level analyses, differentially expressed genes were compared with the GRCh37 list of genes as background; for transcript analyses, differentially expressed gene IDs derived from the transcript were examined using all transcripts identified by RNAseq that met quality control conditions as background; for transcripts with alternatively spliced isoforms, the list was ranked by absolute value of ΔPSI before GO process analysis compared with all differentially expressed transcripts, after assigning transcripts to specific gene IDs. GO Processes with *p*-values < 1.00E-03 were used for constructing graphical and Venn diagrams (http://bioinforx.com/free/bxarrays/venndiagram.php). GO Processes were ranked by –log_10_ *p*-value (pval) for display.

## Results

### RNAseq transcriptome of *A. phagocytophilum*-infected ATRA-differentiated HL-60 cells

**Figure 1:**
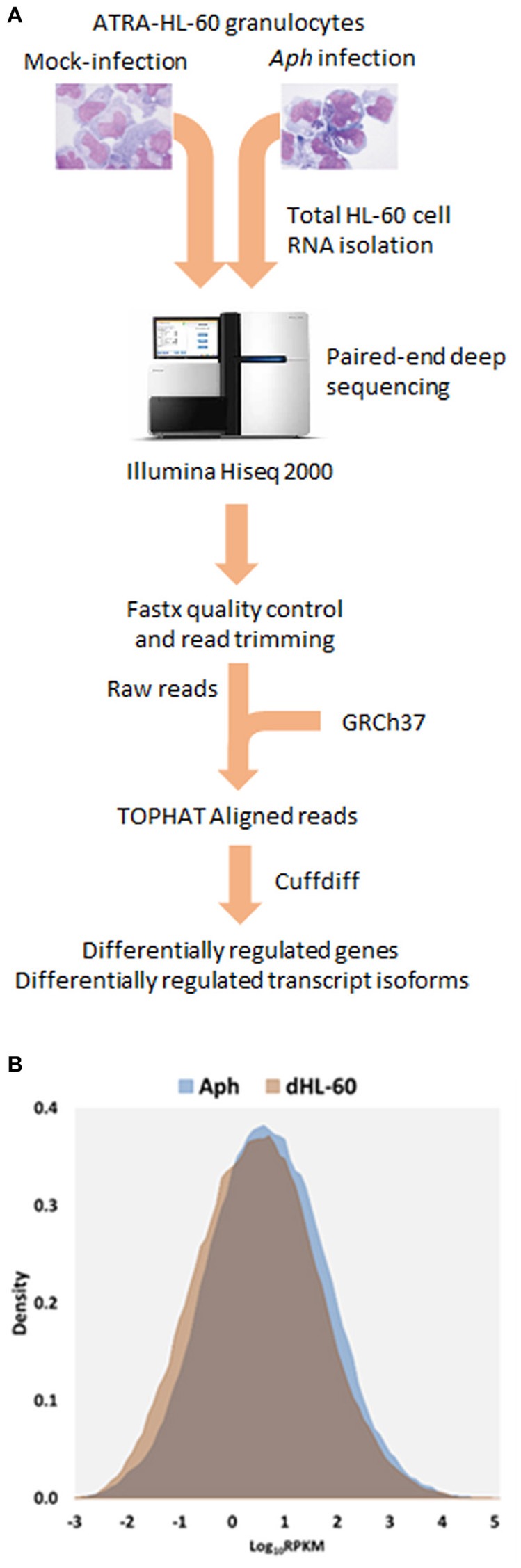
**(A)** Flow-chart of the RNAseq experiments. *A. phagocytophilum*-infected and uninfected ATRA differentiated HL-60 granulocytes were propagated for 5 days until heavily infected; total host RNA was isolated and sent for RNAseq, and then raw reads were assembled after RNAseq, to obtain RPMK (gene level analyses) and FPMK (transcript-level analyses). **(B)** Density plots overlaying distribution of gene level RPKM in *A. phagocytophilum* infected and uninfected ATRA-differentiated HL-60 cells. Note the marked increase in overall gene-level transcript quantities, as noted by a shift in the density plot.

**Figure 2:**
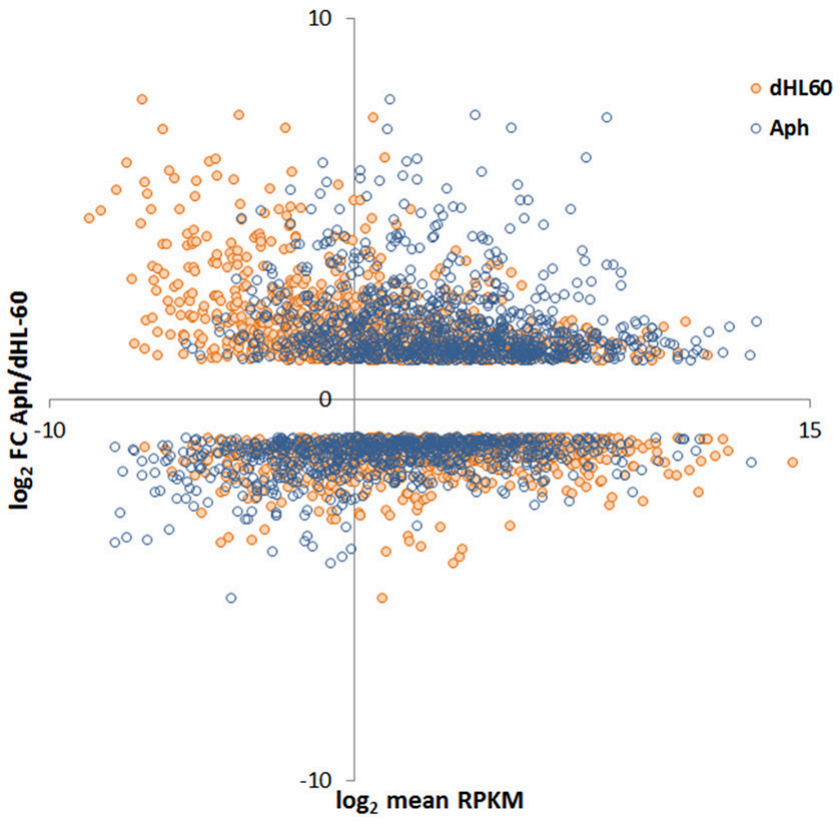
MA-plot of HL-60 cell gene expression in *A. phagocytophilum*-infected vs. uninfected cells. Here, the RPKM values for infected and uninfected cells were plotted against the single log_2_ fold-change value of *A. phagocytophilum*-infected/uninfected cells; thus, each infected/uninfected log_2_ fold value will have two distinct RPKM values. The plot demonstrates a marked shift of upregulated genes to higher RPKM values for *A. phagocytophilum*-infected vs. uninfected cells.

**Figure 3:**
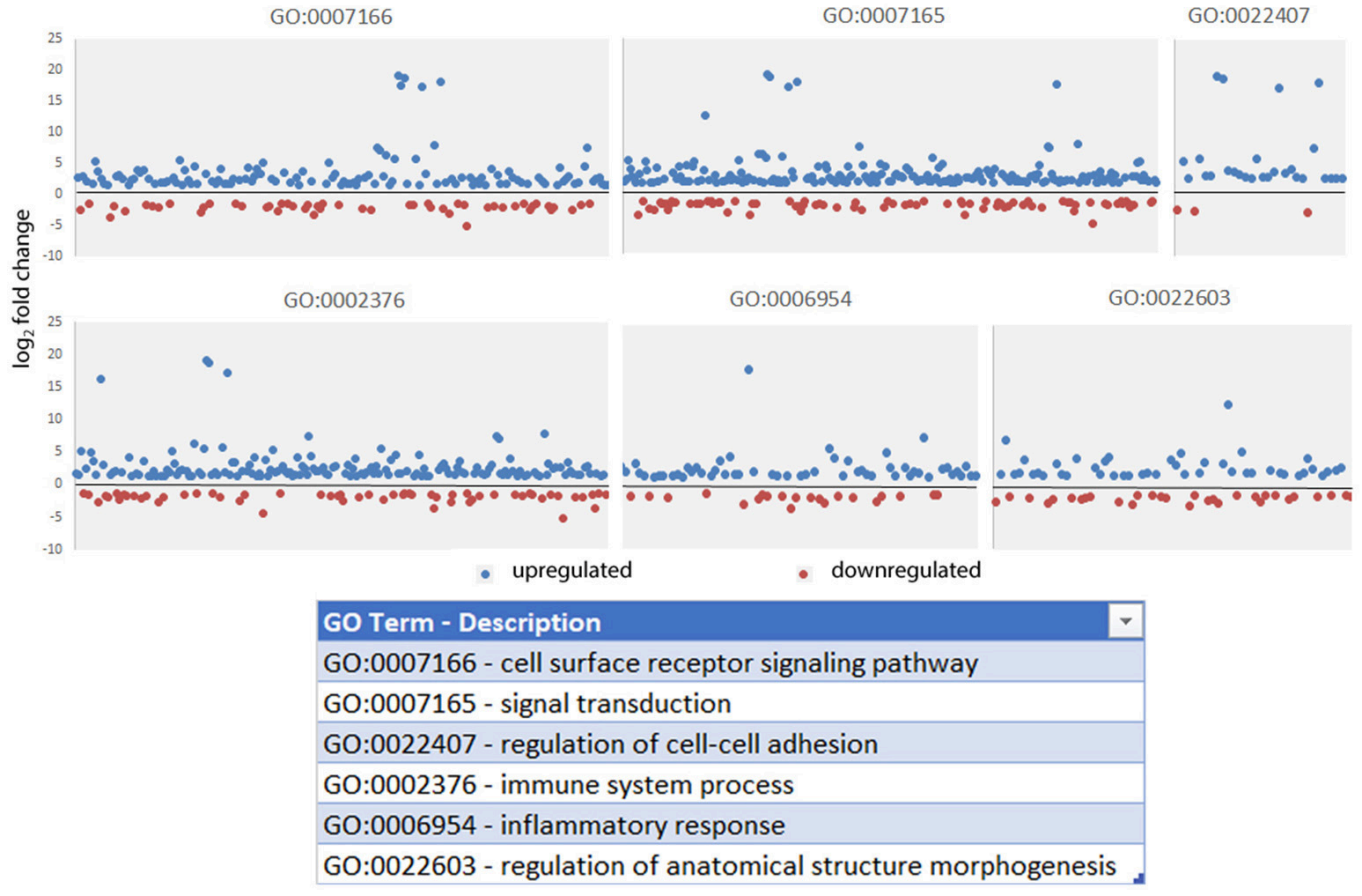
Gene Ontology processes significantly enriched in gene-level analysis of *A. phagocytophilum*-infected ATRA-differentiated HL-60 cells. Here, the absolute value of the log_2_ fold change in gene-level differential transcription was used to generate the list, and representative processes are shown here to demonstrate the range of differential gene transcription over several significantly-enriched processes known to be important for *A. phagocytophilum* survival and fitness.

**Figure 4:**
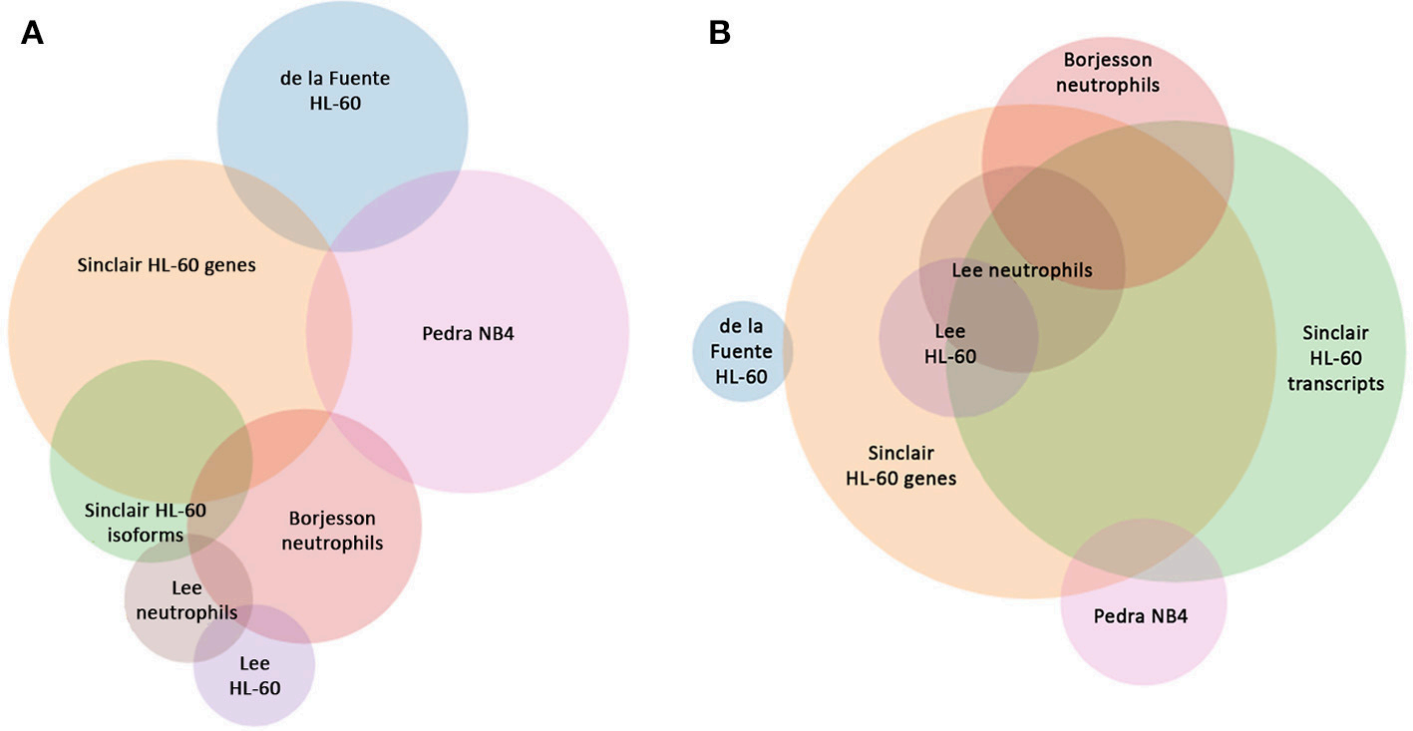
Comparison of shared differentially-expressed genes and GO Processes for *A. phagocytophilum* expression profiling in human myeloid cells and *ex vivo* neutrophils. **(A)** is based upon gene identifiers in each study and demonstrates a lack of overlap in shared differentially-regulated genes in six distinct studies, including five based on microarray analyses, and this study that separately analyzes gene-level and transcript level differential expression in ATRA-differentiated HL-60 cells. **(B)** is based upon GO processes enriched for each study and demonstrates the much greater similarity between studies. Venn circles are labeled by the first author of each publication from which data was derived; here Sinclair represents the data in this manuscript.

Read metrics indicated high quality results for RNAseq, with averages of > 81,000,000 and 89,000,000 reads for infected and uninfected cells, respectively, with >85 and 75% mapped, respectively, and >93.5% mapped to exons in both conditions (Figures S1, S2). A total of 1,740 differentially-expressed genes were identified after QC measures with *A. phagocytophilum* infection, including 968 upregulated and 772 downregulated more than 2-fold compared to uninfected ATRA-differentiated HL-60 cells. To assess the overall change in gene-level transcript reads between infected and uninfected cells, we examined the number of gene-level transcripts in RPKM infected and uninfected cells and found there was a marked increase in transcripts with infection (Figure 1). Similarly, when the number of gene transcripts in infected and uninfected cells was examined with respect to differential transcription, there was an increase in transcript quantities among upregulated genes with infection (Figure 2). To determine which biological pathways were most impacted by the changes in gene-level transcription, we conducted GO process analysis using a set of 1353 genes recognized within the GO gene term sets. A total of 64 GO processes were enriched with *p*-values ranging from < 0.001 to 1.16 × 10^−7^, and ranging in enrichment from 1.07 to 1353. As anticipated from prior *A. phagocytophilum* transcriptome studies in human myeloid cells, processes identified with GO analysis included cell surface receptor signaling pathways, processes related to immune system and interferon-gamma-mediated signaling, to antigen processing via MHC and regulation of apoptosis/cell death (Figure 3). Compared to other expression profiling studies that examined myeloid cell gene expression with *A. phagocytophilum* infection by microarray, there was only a small proportion of differentially expressed genes shared among any of the studies (Figure 4A), suggesting that a core set of genes are likely to regulate most functions in response to or controlled by *A. phagocytophilum*. However, since most studies identified similar altered transcriptional programs, GO analysis and comparison of enrich GO processes with each study showed a higher degree of similarity for all but the studies of de la Fuente et al. (2005) (Figure 4B).

### *A. phagocytophilum* infection alters the expression of alternative splicing events in cellular transcripts

**Figure 5:**
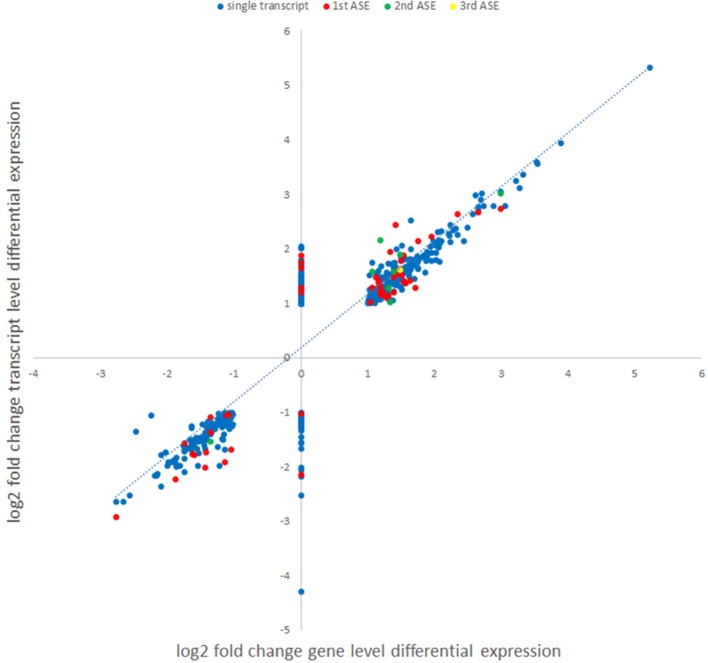
Comparison of gene level and transcript-level changes in expression between *A. phagocytophilum*-infected and uninfected ATRA-differentiated HL-60 cells. Each identified differentially-expressed transcript log_2_ fold change value was used to map the log_2_ fold change value of expression identified in the gene-level analysis. Alternative splicing events identified in the isoform analyses are labeled in different colors (blue, single or main transcript; red, first ASE; green, second ASE; yellow, third ASE). Genes lacking identified significant differential gene expression (assigned log_2_ fold change value = 0) for which differential transcription was identified at the isoform-level analysis.

**Figure 6:**
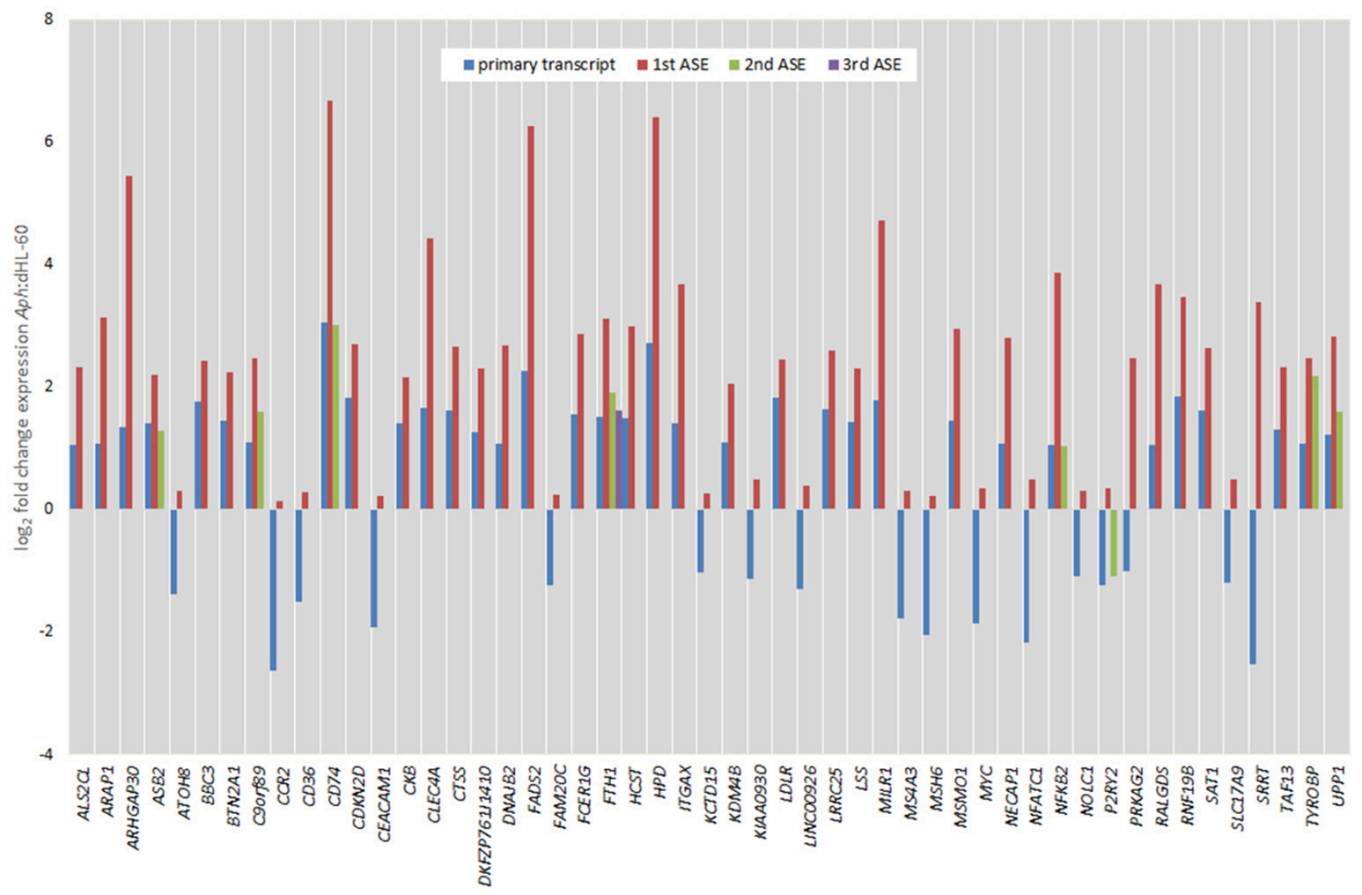
Comparison of fold change expression levels among alternative splicing events for 51 genes identified by transcript-level RNAseq. The primary transcript is shown in red, with ASEs depicted by blue, green, or yellow bars.

**Figure 7:**
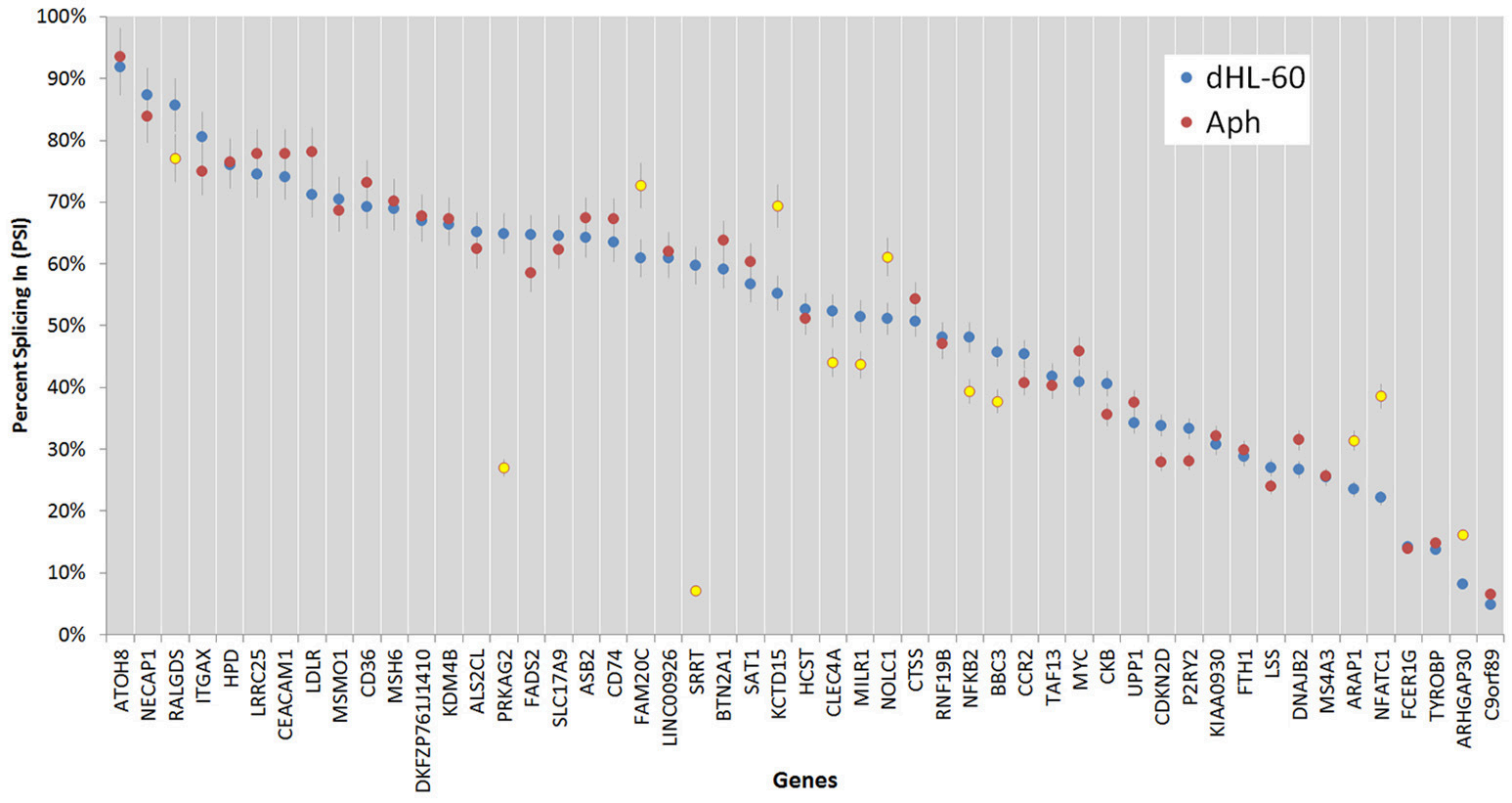
Percent splicing in for the alternative spliced events was calculated as described in the methods. The PSI distribution is shown for all ASEs; blue markers represent PSI values for uninfected ATRA-differentiated HL-60 cells; red markers show the PSI values for *A. phagocytophilum*-infected cells, with those deviating from the HL-60 PSI values by more than 7.5% highlighted in yellow; error bars represent 7.5% change range by comparison with infected or uninfected cells.

In order to examine whether an alternative cellular transcriptional landscape of infection by *A. phagocytophilum* occurs, the RNAseq data were evaluated for the presence of alternative transcript isoforms and compared with gene-level analyses. As with the gene-level analyses, only transcripts with absolute differential expression >2 fold between *A. phagocytophilum*-infected and uninfected ATRA-differentiated HL-60 cells were evaluated. Overall, 195,553 transcript reads were detected either in infected or uninfected cells, including transcripts from 59,747 unique genes or annotated gene features. After filtering for QC and significant fold change expression, but not FPKM cutoff, a list of 1,075 isoforms from 958 unique genes was identified, including 862 with one differentially expressed isoform, 77 genes with 2 differentially expressed isoforms, 17 genes with 3 differentially expressed isoforms, and 2 genes with 4 differentially expressed isoforms. Among the 96 genes with 2 or more differentially expressed isoforms, 60 genes were upregulated for all isoforms, 30 genes were downregulated for all isoforms, and 6 genes showed isoforms that were both up- and down-regulated. When the list was further filtered to include those with at least 1 FPKM, a total of 665 transcripts from 605 unique genes were identified, including 111 distinct transcripts representing alternatively spliced isoforms from 51 distinct gene loci. Gene-level differential transcription was similar to that found with evaluation at the transcript level, when overall differential expression was calculated for isoforms found in those 51 gene loci (Figure 5). However, as in other studies of viruses and bacteria, RNAseq transcript isoforms yielded a distinct profile for transcriptional expression that supports the conceptual role of alternative splicing in control over host transcriptional programs and functions. Thus, PSI was calculated for each of the ASEs associated with specific genes, and examined in more detail for those with ΔPSI >7.5%. In this analysis, 51 genes had two distinct differentially expressed transcripts, 8 had 3 differentially expressed transcripts, and 1 had 4 differentially expressed transcripts (Figures 6, 7).

**Figure 8:**
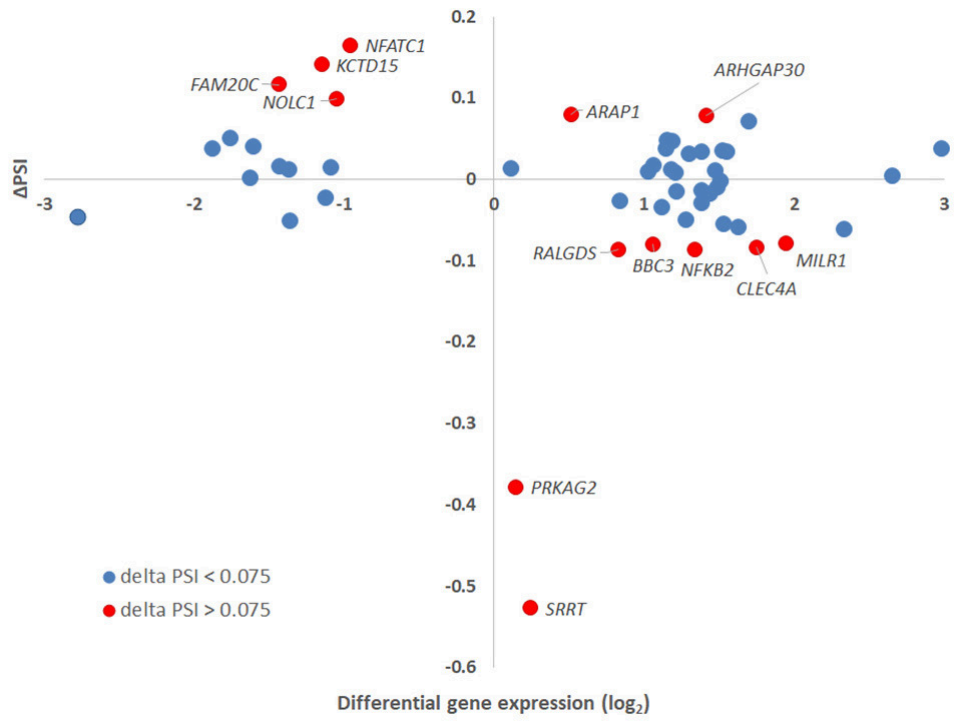
Delta PSI and gene-level differential expression. The absolute values of PSI for isoforms assigned to a specific gene were plotted against the gene-level differential expression in *A. phagocytophilum*-infected vs. uninfected ATRA-differentiated HL-60 cells. Thirteen genes had ΔPSI greater than 7.5%, but only eight had gene-level differential expression greater or less than 2-fold. Of the three genes with the greatest ΔPSI, none had gene-level differential expression changes greater or less than 2-fold, but at least one isoform for each of these was expressed at 2-fold greater or 2-fold lower levels than in uninfected cells.

Of all ASEs, differential transcription varied considerably from marked upregulation, e.g., with *CD74* and *FADS2* to moderate downregulation, as with *SRRT* and *CCR2*. Of the 51 genes with ASEs, for 34, all ASEs were upregulated and for 15, all ASEs were downregulated; however, 2 genes had both up- and downregulated splicing events—as much as a 20-fold difference in isoform expression for *SRRT*, for which the gene product is believed to be involved in microRNA processing and in transcript splicing events (Figure 6). Similarly, for each gene, alternative splicing events were examined to generate the PSI metric for comparison of infected and uninfected cells, where large deviations (defined as >7.5% deviation from uninfected cell PSI) could indicate impacted processes in part regulated with *A. phagocytophilum* infection (Figure 7). Among the 51 genes with ASE events, the PSI for 13 varied from that of uninfected HL-60 cells by >7.5%, including seven for which PSI was less with infection, and 6 for which PSI was greater. For five genes, PSI was more than 10% changed (*SRRT*, −53%; *PRKAG2*, −38%; *NFATC1*, +16%; *KCTD15*, +14%; and *FAM20C*, +12%). Among the 13 genes for which ΔPSI was > 7.5%, differential gene-level expression was significantly lower or higher in infected cells for 8, although these had ΔPSI values below 15%, whereas the three genes with the greatest ΔPSI did not demonstrate gene-level differential transcription; yet, for each of two detected isoforms, at least one demonstrated significant differential expression (Figure 8).

### Transcript isoform length and spliceosome

Other investigators found that the average length of ASE transcripts decreases with infection. Figure S1 shows the relationship between transcript length for those significantly transcribed isoforms with more than one isoform detected vs. those transcripts for which only a single isoform was identified. There were no significant differences identified in transcript length or in fold-change gene expression associated with transcript length (*p* = 0.114; Student's *t*-test) when ASEs were compared with single transcripts. Since 51 of 606 genes (8%) in gene-level RNAseq and 111 transcripts were identified in transcript-level RNAseq from those 606 genes, it was evident that the spliceosome and its components were likely involved, as observed with viral and *M. tuberculosis* infections. Thus, expression of genes encoding components of the spliceosome were examined at gene- and isoform-levels to determine whether alternative splicing of transcripts encoding spliceosome components could also be impacted by this process. At the gene level, none of the 135 genes in the sliceosome list were identified as differentially regulated; however, when the list of differentially transcribed isoforms was examined, 9 isoforms were identified spanning all components of the sliceosome complex, and with the exception of *HNRNPC* (−3 log_2_-fold), all were upregulated with infection at an average of 4.7 log_2_-fold (range 3.2- to 6.1 log_2_-fold) (Table S1).

### Gene ontology analysis of genes, transcripts, and ASEs

**Figure 9:**
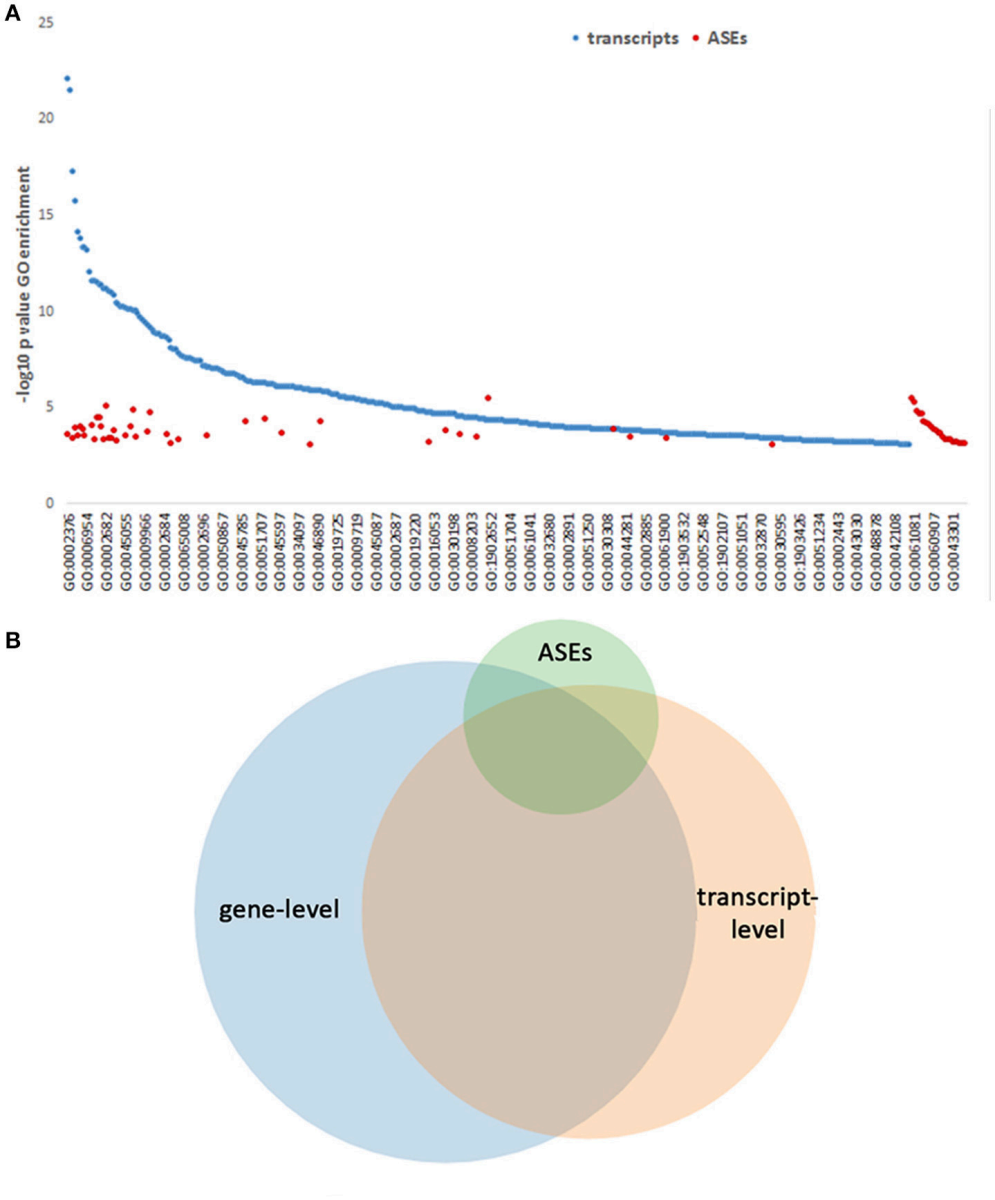
GO Process predictions based on gene-level, transcript-level, and ASE analyses. GO Processes were queried and those with *p*-values < 0.001 for each of the above categories are displayed ranked by –log_10_ *p*val for display. **(A)** GO Processes identified by transcript analysis span a wide range, with some overlap of those GO Processes identified by ASE only analysis, which predicts additional pathways. The full list of GO processes represented on the x-axis is included in Table S2. **(B)** Venn diagram showing the overlapping GO Processes identified by gene-level, transcript-level, and ASE-level analyses, demonstrating a unique set of GO Processes associated with ASEs.

To determine whether separate functions could be regulated or directed by the ASEs promoted with infection, all gene-level identifications, transcripts and separately ASEs were examined for enrichment in GO process analysis. This identified 430 GO Processes associated with differentially expressed genes at *p*-values < 0.001. When differentially expressed transcript gene IDs were examined as a single list ranked by absolute value log_2_-fold change in expression, GO analysis identified 53 processes, which have relevance for *A. phagocytophilum* fitness (Tables S2, S3). Owing to the inclusion of background sets for some, GO process analysis focusing on all differentially expressed transcript isoforms (using all detected transcripts as background) and ASEs sorted according the absolute ΔPSI (using all differentially expressed transcript isoforms as background) revealed larger sets of GO processes: 393 for differentially expressed transcripts and 64 for differentially expressed ASEs; 84 GO processes were identified in both the differential transcript and ASE evaluations. For all differentially expressed transcripts, these focused on host defense, inflammatory response, respnose to external stimulus, cytokine-mediated signaling, signal transduction regulation, vesicle transport, programmed cell death regulation, chemotaxis, and related subprocesses; with ASE enrichment, GO analysis revealed shared pathways, including those specific for neutrophils (neutrophil activation in immune response, degranulation, regulated exocytosis, leukocyte migration, regulation of myeloid leukocyte differentiation) and pathways not shared (including cellular response to low-density lipoprotein particle stimulus, intestinal lipid absorption, intestinal cholesterol absorption, sterol import, cholesterol import, positive regulation of myeloid leukocyte cytokine production involved in immune response, positive regulation of macrophage cytokine production, positive regulation of cytokine production involved in immune response, regulation of blood coagulation, regulation of hemostasis, regulation of coagulation, among others) (Figure 9A and Table S2). In fact, owing to the related nature of GO processes in myeloid and bone marrow-derived cells, biological processes predicted by GO often share gene products that have overlapping or multiple functions (Figures 9A,B); thus, significant enrichment for similar processes is observed by GO processes identified separately by gene-, transcript-, and ASE-level analyses. Overall, many but not all GO Processes were shared among gene-, transcript-, and ASE-level analyses, and each analysis identified uniquely enriched GO Processes not significantly enriched otherwise (Figure 9B; Table S2; Figure S2).

## Discussion

In the post-genomic era, a major incongruency between the numbers of genes in the human genome and the number of protein products is widely recognized (Wang et al., 2008). There are two major processes by which the eukaryotic proteome is expanded from that encoded by single genes in the genome, including alternative splicing and alternative translation initiation signals (Miles et al., 2017). For the former, pre-messenger RNA processing in eukaryotes results in the production of several or many mRNA variants from a given gene (Baralle and Baralle, 2017). While this premise indicates that alternative splicing from individual genes generates isoforms and functional diversity, the overall impact of alternative splicing and its effects on regulating and tuning gene networks is now increasingly studied. Alternate splicing clearly plays a critical role in generating biological complexity and is thereby subject to dysregulation as observed with abnormalities in the spliceosome as associated with some human diseases (Barash et al., 2010), or in development (Keightley and Lieschke, 2017), cancer (Narayanan et al., 2017), and other inherited disease processes (Ramanouskaya and Grinev, 2017). The roles that such alternate splicing events play after exposure to or under the direct influence of infectious agents, in particular microbes and viruses for which the eukaryotic genome, transcriptome, and metabolome are extensions of the agent's genomic repertoire, is in particular underappreciated (Boudreault et al., 2016; Kalam et al., 2017; Wang et al., 2017).

While viral infection is well-known to promote alternative splicing as a mechanism for subversion of host processes, improvement of viral fitness, and in pathogenic outcomes, the extent to which alternative splicing occurs in both viruses, and more recently with intracellular bacterial infection, is at minimum abundant and of potentially great impact toward understanding pathogenicity (Akusjarvi, 2008; Boudreault et al., 2016; Hu et al., 2016; Graham and Faizo, 2017; Kalam et al., 2017; Wang et al., 2017). *M. tuberculosis* infection pathogenicity directly relates to alternative splicing events that vary with time after infection of cultured macrophages, leading to transcription of some isoforms for which premature stop codons are present that preclude translation and result in early degradation through nonsense mediated decay (Kalam et al., 2017). Other isoforms exclude or include one or more exons, providing new contributions to the proteome each with a potentially distinct functional outcome.

Here we demonstrate that by using next generation RNAseq methods that can identify the presence and quantity of mRNA isoforms, not achievable with microarray analyses previously used to discern expression pattterns of human genes with *A. phagocytophilum* infection, that there is extensive global alternative splicing. The methodology we used has some advantages and disadvantages: infection of human neutrophils *ex vivo* by *A. phagocytophilum* is challenging and the mere manipulation of neutrophils *ex vivo* presents a variety of obstacles including achieving infection among even a small proportion of cells for which the whole population is used in average to estimate transcriptional responses to active infection (Borjesson et al., 2005; Lee et al., 2008; Rennoll-Bankert et al., 2014). To overcome this barrier, we and others have employed myeloid model cell lines that allow high levels of infection to be achieved, but suffer from potential confounding experimental outcomes owing to genomic rearrangements, deletions, or aneuploidy of cells like HL-60 and NB4 promyelocytic leukemia cells, PLB-985 acute myelomonoblastic leukemia cells, or THP-1 acute monocytic leukemia cells (Goodman et al., 1996; Pedra et al., 2005; Garcia-Garcia et al., 2009a; Rennoll-Bankert et al., 2014). We previously showed that the use of ATRA-differentiated HL-60 cells yields a transcriptional profile like that of infected neutrophils that are sorted to high proportion of infection than could be achieved with bulk culture alone (Rennoll-Bankert et al., 2014). Using this approach, we identified the transcriptional responses of *A. phagocytophilum*-infected ATRA-differentiated HL-60 cells compared to mock-infected cells as a basis for advanced studies of gene regulation in the context of infection, including the epigenome and how *A. phagocytophilum*-exported DNA-binding proteins impact gene transcription and nuclear architecture.

Through the process of RNAseq, we established both gene-level transcriptional responses, identifying 1,740 differentially expressed genes, and these genes predicted similar gene ontology processes as observed using microarray transcriptome analysis in primary human neutrophils and any of the myeloid cell types able to sustain infection. In contrast, the algorithms used to identify differentially expressed transcript isoforms yielded a smaller number overall-−605 gene IDs among a total of 665 total differentially expressed transcripts, including 51 ASEs with 111 unique transcripts, including 8 with 3 distinct isoforms, and 1 with 4 isoforms that were differentially expressed. That 18% of all differentially expressed genes identified at the transcript isoform level with FPKM >1 included alternative splicing events was a surprising finding, but consistent with the emerging literature with virus and *M. tuberculosis* studies. Our study was limited in that whether these differentially expressed ASEs were previously identified in neutrophils was not examined.

The *M. tuberculosis* and viral infection studies underscore several features of interest, which we pursued (Boudreault et al., 2016; Kalam et al., 2017). For *M. tuberculosis*, an increase in the expression of truncated and non-translatable isoforms was found, especially with more virulent infections, and included a significant regulation of the macrophage spliceosome components that presumably belie these events. This obervation is an example of how controlling key checkpoints or master regulators can have a very substantial effect on the biology of the infected cells and the microbe (Sinclair et al., 2014). For viral infections, a variety of global changes, of similar magnitude as seen here with *A. phagocytophilum* infection, also occur, but unlike for *M. tuberculosis*, there are only limited changes in expression or splicing patterns of splicing factors, except for the overexpression of an alternative transcript from the splicing regulator *ESRP1* (Boudreault et al., 2016). Another observation shows that *M. tuberculosis* infection of human macrophages yields overall shorter transcripts associated with ASEs and that this is associated with reduced translation into proteins. A similar phenomenon was not identified with *A. phagocytophilum*, where the average transcript size of the alterative isoforms did not vary significantly from uninfected to infected conditions. Similarly, of the 136 genes most involved in the human spliceosome, considerable alternative splicing events in this group were not noted with virus or with *A. phagocytophilum* infection. However, with *A. phagocytophilum* infection of differentiated HL-60 cells, in the transcript analysis (but not in the gene-level analysis), there was marked differential expression of spliceosome transcripts that potentially impact each of the critical spliceosome stages. Whether such differential expression impacts the high degree of alternative splicing observed is not known, but is under study.

Perhaps of greatest interest is how the differentially-expressed alternative spliced transcripts or transcript gene IDs not detected as differentially regulated with gene-level analysis, predict altered cellular processes that govern cell function. *A. phagocytophilum* infection has a profound impact on neutrophils including alterations at virtually every cardinal funtion (Dumler, 2012). Many of these changes can be documented to be in part regulated at the level of gene transcription (Sinclair et al., 2014). For some of these changes a direct regulatory role has been established for the *A. phagocyotphilum* type 4 secretion system effector AnkA, while other secreted effectors have a greater impact by protein-protein interactions in signaling, cytoskeletal alterations, or endosomal trafficking (Carlyon and Fikrig, 2006; Rikihisa, 2011; Truchan et al., 2013; Rennoll-Bankert et al., 2015). While no apparent direct association of AnkA with the ASEs was observed (data not shown), *A. phagocytophilum* significantly regulates DNA methylation, in particular at intron and exon junctions, and DNA methylation is posited as an important mark for slowing of RNA polymerase and induction of alternative splicing (Shukla et al., 2011; Maunakea et al., 2013; Sinclair et al., 2015). Likewise, no specific colocalization of meDNA and ASEs was established here.

Reprogramming of cellular functions can be a challenge to study, and surveys of process changes can be conducted using bioinformatic tools such as gene ontology (GO) analysis. While the GO analysis at the gene- and transcript-levels identified core shared processes enriched during *A. phagocytophilum* infection of differentiated HL-60 cells, there remained a unique set identified solely by one or the other method of differential gene expression analysis. Likewise, the analysis of the smaller 51 gene, 111 transcript set of alternatively spliced isoforms identifies both shared and unique sets of GO processes, the latter predominantly focused upon lipids, lipoproteins, and cholesterol/sterol metabolism, but also including regulation of cytokine production and neutrophil degranulation, all recognized as critical events for *A. phagocytophilum* fitness leading to enhanced intracellular survival and spread (Dumler, 2012). That the regulation of lipids and lipoproteins was somewhat selectively enriched when ASE differentially expressed genes were examined suggests a more specific role in these processes, for which recent investigations have shown a critical role for cholesterol (Manzano-Roman et al., 2008; Xiong and Rikihisa, 2012), phospholipid and eicosanoids (Wang et al., 2016), as well as lipoproteins and polar lipids (Choi et al., 2004b; Choi and Dumler, 2007) which can directly or indirectly affect proinflammtory state in neutrophils that results in cytokine production and initiation of innate and adaptive immune response, including inflammasome triggering (Dumler et al., 2000, 2007; Martin et al., 2000; Wang et al., 2016). Of course, in silico analyses leave great overlaps and suggest cross-talk because the GO processes identified by each approach applied here share many genes. Although little investigation has been conducted of how infection affects gene regulation at the level of pre-messenger RNA processing and splicing, this work indicates that ASEs are a significant comoponent of the transcriptional response to *A. phagocytophilum* infection. Yet, those attributes of ASEs identified for viral and *M. tuberculosis* infections are not entirely shared with observations of ASEs in *A. phagocytophilum* infection. This provides evidence that a diversity of responses to infectious agents can further focus and refine the host cell proteome. How these events inform the separate aspects of microbial fitness in the unusual niche of a neutrophil vacuole, from the ability to sense microbial infection, such as via the inflammasome, to eliciting inflammatory injury and immune dysregulation will be clearly more complex than anticipated, and will be an area for intense investigation over time.

## Data availability

The datasets generated and analyzed for this study can be found in the Gene Expression Omnibus database with GEO Accession GSE107770 (https://www.ncbi.nlm.nih.gov/geo/query/acc.cgi?acc=GSE107770).

## Author contributions

JSD helped to design the project, coordinated experimental and analytical approaches, analyzed data and wrote the manuscript. SS conceived the project, designed the experimental project, executed the experiments and coordinated the library preparations and the sequencing. AS conducted primary bioinformatics processing and quality control of the sequencing data, conducted the majority of the identification of RNA isoforms, and provided statistical analysis for quality control purposes. All authors contributed to writing and revisions of the manuscript.

### Conflict of interest statement

J. Stephen Dumler: The opinions expressed herein are those of the author(s) and are not necessarily representative of those of the Uniformed Services University of the Health Sciences (USUHS), the Department of Defense (DOD); or, the United States Army, Navy, or Air Force.

The other authors declare that the research was conducted in the absence of any commercial or financial relationships that could be construed as a potential conflict of interest.

## Supplementary material

Figure S1 Among all isoform transcripts that met quality control measures for both infected and uninfected ATRA-differentiated HL-60 cells, isoform length in bases was plotted against differential isoform expression to test the hypothesis that alternative splicing events result in shorter isoforms. Among those genes with more than one isoform detected compared to single isoforms, there was a range of both isoform lengths and differential expression, but differential expression was not significantly linked with isoform length.

Figure S2 Enrichment of GO Processes and the significance of the enrichment for all differentially expressed isoforms and for a set limited to alternatively-spliced isoforms. Enrichment and *p*-value were derived from the enrichment profile of the GO analyses.

Table S1 Human spliceosome components examined for differential gene expression by gene-level and transcript isoform-level RNAseq.

Table S2 GO Process enrichment for all transcripts and alternatively spliced isoforms (ASE).

Table S3 Panther GO-Slim Biological Processes associated with alternatively spliced isoforms after A. phagocytophilum infection of ATRA-differentiated HL-60 cells.
