# Supplementary figures and images for: Alternative Splicing of Differentiated Myeloid Cell Transcripts after Infection by Anaplasma phagocytophilum Impacts a Selective Group of Cellular Programs
Source: Front Cell Infect Microbiol. 2018 Feb 2;8:14. doi: 10.3389/fcimb.2018.00014 (PMC5801399; doi:10.3389/fcimb.2018.00014)

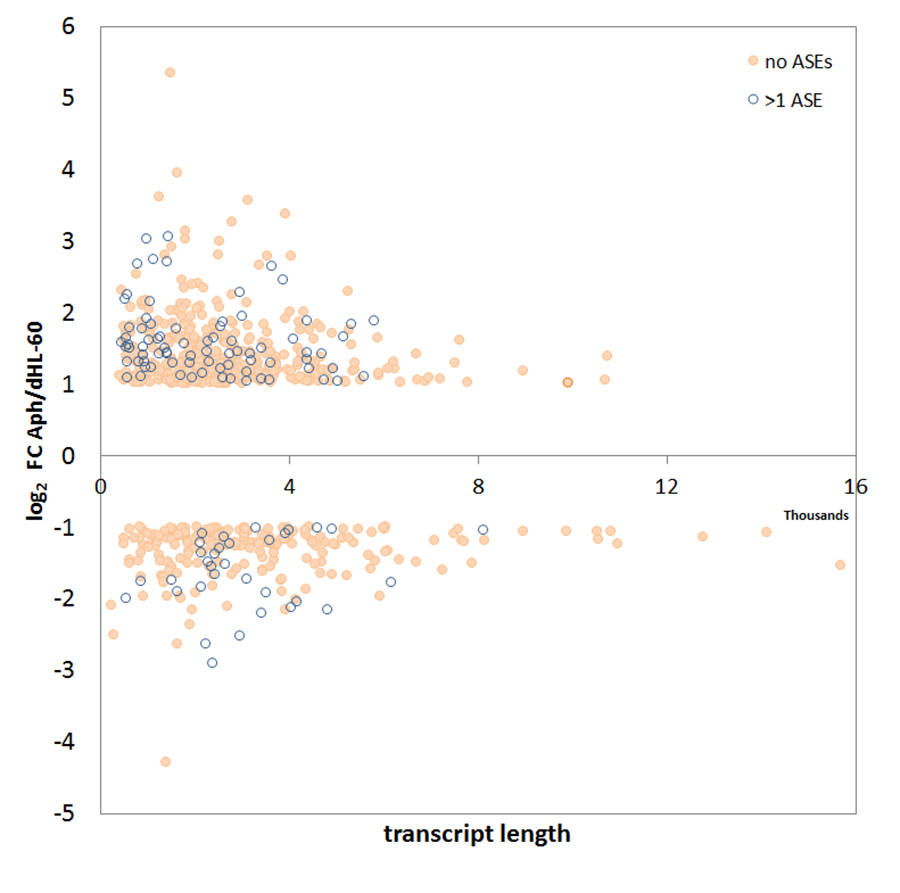

Supplement: Figure S1 — Among all isoform transcripts that met quality control measures for both infected and uninfected ATRA-differentiated HL-60 cells, isoform length in bases was plotted against differential isoform expression to test the hypothesis that alternative splicing events result in shorter isoforms. Among those genes with more than one isoform detected compared to single isoforms, there was a range of both isoform lengths and differential expression, but differential expression was not significantly linked with isoform length. [file Image1.JPEG]

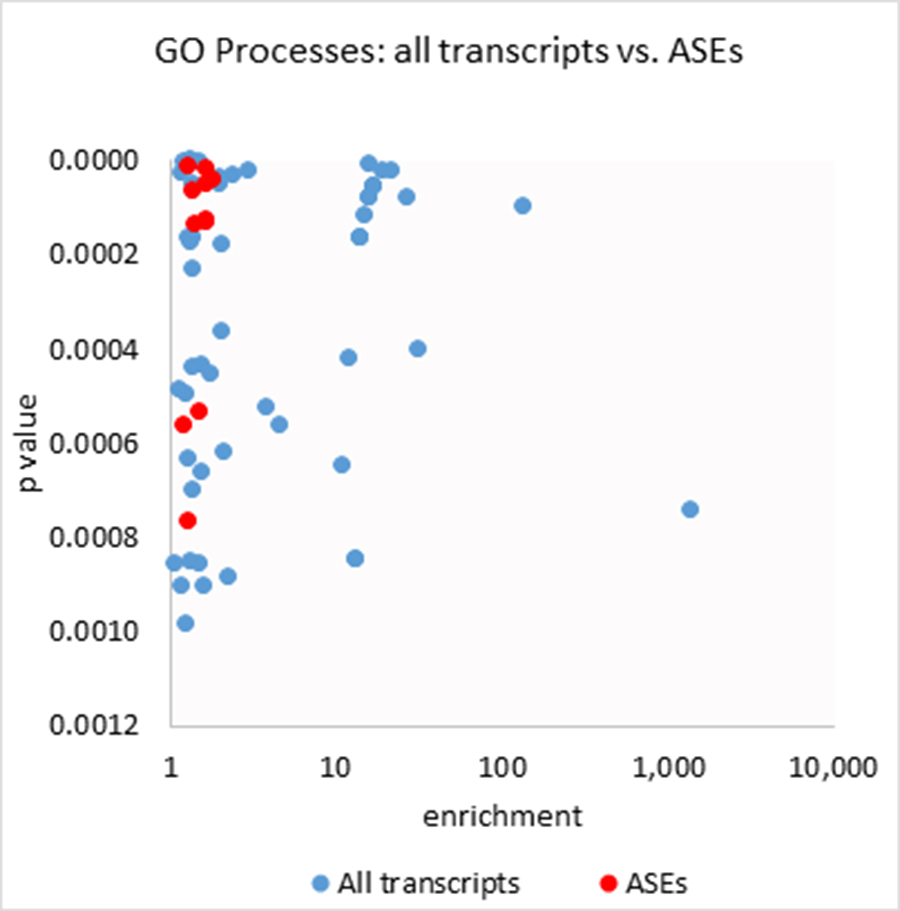

Supplement: Figure S2 — Enrichment of GO Processes and the significance of the enrichment for all differentially expressed isoforms and for a set limited to alternatively-spliced isoforms. Enrichment and p-value were derived from the enrichment profile of the GO analyses. [file Image2.JPEG]
